# P348: Medical emergencies in national hospital of Lamorde, Niger

**DOI:** 10.1186/2047-2994-2-S1-P348

**Published:** 2013-06-20

**Authors:** H Djibo, KK Issa, M Arzika, AI Toure

**Affiliations:** 1 National Hospital of Niamey, Niamey, Niger

## Objectives

The emergency department of the National Hospital Lamordé is the venue for all patients whose care has not been programmed. But its use as a gateway to the health care system generates a space which decreases efficiency for real emergencies. Identifying the causes of malfunctions is essential to start making provision for improved organization and provision of emergency services.

## Methods

We conducted a prospective cross-sectional study in the emergency department of the National Hospital Lamordé from 1 January to 31 December 2005. The survey made it possible to follow, 780 patients treated for medical and pediatric problems using individual records. Our variables are socio-demographic, clinical, paraclinical and therapeutic.

## Results

The mean age of patients was 24.8 years with a female predominance of 57%, 69.2% of visits are made to the hours of service the average waiting time is 20min consultations with 90min peak rush hour, 100% of transport were not medicalized and 97.6% were provided by untrained people, 76% of patients have made a self-reference, the majority or 72% of consultations were performed by students, 44% of cases one needs urgent care, pediatric emergencies represent 25.4% of admissions, malaria is the leading cause of under observation with 62.85% of cases, the completion rate effective diagnostic tests is 46.6%. These results come after a mean waiting time of 90min, the average waiting time is 30min care; 95.9% of patients made comments emergencies had a favorable, the mortality rate was 41%. Coma and PCBs are the primary causes each with 25% of cases and malaria is the leading cause of mortality of pediatric cases with 27.14% of cases.

## Conclusion

The finding results inspired our recommendations for improving the organization, the service efficiency and quality of structures by conventional standards.

## Disclosure of interest

None declared

